# Towards Automated Large-Scale 3D Phenotyping of Vineyards under Field Conditions

**DOI:** 10.3390/s16122136

**Published:** 2016-12-15

**Authors:** Johann Christian Rose, Anna Kicherer, Markus Wieland, Lasse Klingbeil, Reinhard Töpfer, Heiner Kuhlmann

**Affiliations:** 1 Institute of Geodesy and Geoinformation, Department of Geodesy, University of Bonn, Nussallee 17, 53115 Bonn, Germany; wieland@igg.uni-bonn.de (M.W.); klingbeil@igg.uni-bonn.de (L.K.); heiner.kuhlmann@uni-bonn.de (H.K.); 2 Julius Kühn-Institut, Federal Research Centre of Cultivated Plants, Institute for Grapevine Breeding Geilweilerhof, 76833 Siebeldingen, Germany; anna.kicherer@julius-kuehn.de (A.K.); reinhard.toepfer@julius-kuehn.de (R.T.)

**Keywords:** viticulture, field phenotyping, 3D point cloud, multi-view-stereo, classification, berry diameter, number of berries, number of grape bunches

## Abstract

In viticulture, phenotypic data are traditionally collected directly in the field via visual and manual means by an experienced person. This approach is time consuming, subjective and prone to human errors. In recent years, research therefore has focused strongly on developing automated and non-invasive sensor-based methods to increase data acquisition speed, enhance measurement accuracy and objectivity and to reduce labor costs. While many 2D methods based on image processing have been proposed for field phenotyping, only a few 3D solutions are found in the literature. A track-driven vehicle consisting of a camera system, a real-time-kinematic GPS system for positioning, as well as hardware for vehicle control, image storage and acquisition is used to visually capture a whole vine row canopy with georeferenced RGB images. In the first post-processing step, these images were used within a multi-view-stereo software to reconstruct a textured 3D point cloud of the whole grapevine row. A classification algorithm is then used in the second step to automatically classify the raw point cloud data into the semantic plant components, grape bunches and canopy. In the third step, phenotypic data for the semantic objects is gathered using the classification results obtaining the quantity of grape bunches, berries and the berry diameter.

## 1. Introduction

Grapevine is a perennial crop, and therefore, phenotypic evaluations of yield traits need to be done directly in the field. Vineyards typically encompass large areas that contain thousands of single grapevines, with each of these grapevines being able to possess a slightly different phenotype. In grapevine breeding, the screening of large sets of substantially different genotypes is a special requirement. Large sets of breeding material are to be screened in their entirety to gather phenotypic data to be used in the breeding program. Traditionally, phenotypic evaluations on the plant organ level are either done by visual estimations or destructive sampling. Both methods are time consuming, often subjective and need to be done by experienced employees. Due to these reasons, the number of samples is often limited. The development of efficient and objective phenotyping techniques and high-throughput field phenotyping platforms is crucial to overcome the phenotypic bottleneck [1].

Yield is one of the most commonly-measured and most complex phenotypic traits in viticulture [2]. It is defined as the crop weight per vine whereby the weight is dependent on the variation of the number of bunches per vine (60%), the number of berries per bunch (30%) and the berry size (10%) [3]. Due to a high variation in seasonal yields, forecasting and controlling yield are major emphases in viticulture. The true biological yield, described as weight per vine, is therefore statistically forecast through assessing the so-called yield parameters: bunch weight, number of bunches, number of berries per bunch and berry weight. Berry size further serves as an important indicator for aroma-related traits, e.g., for sugar content [4]. These are determined mostly destructively from a subset either at the onset of veraison [5] or at harvest [6]. The statistical reliability of the forecast then grows with the amount of data collected to derive the yield parameters. A non-destructive, sensor-based field application to derive yield parameters is therefore of high interest for precision viticulture.

In recent years, several 2D image-based approaches to detect grape bunches [6,7,8,9], berry diameter [10,11] or number [3,12] have been developed. While producing good results, 2D approaches are prone to occlusion and do not usually cover the whole canopy due to the difficulty of registering multiple cameras automatically. In the meantime, the demand for access to the plants’ 3D geometry has become of significant importance for phenotyping in general. Several solutions under laboratory and field conditions employing laser scanning [13,14], time of flight cameras [15,16] structured light [17] or multi-view-stereo (MVS) approaches [18,19,20] are opening the door to 3D phenotyping. Multi-view-stereo approaches are especially suited for reconstructing the complex morphology of grapevines. Using 2D images alone from almost any camera [21], colored 3D points are reconstructed. The method gained popularity due to its adaptability to a multitude of scenarios, its budget friendliness and high detail point clouds. Industrial cameras, equipped with interfaces for automation and triggering procedures, enable the design of more sophisticated sensor systems, which gather images from multiple cameras and perspectives at single point in time [22]. Using multiple perspectives and camera heights greatly reduces the effect of occlusion and therefore enhances data completeness. Furthermore, the access to 3D data holds the potential to derive additional phenotypic parameters.

In recent years, only a few 3D phenotyping approaches have been published in viticulture. Dey et al. [23] used an MVS algorithm on images manually collected with a consumer camera to reconstruct colored point clouds of grapevine rows. The point cloud was then classified into the classes grape bunches, leaves and branches using support vector machines and geometric features based on principal component analysis, as well as color features. They reached a classification accuracy of 96%–98%. As their focus lays on point cloud classification, no comments on the effectiveness of the data acquisition were made, and the point clouds were arbitrarily scaled. No phenotypic data were derived.

Herrero-Huerta et al. [20] constructed point clouds of grapevines using their own MVS software and images of slightly defoliated grapevines taken at daytime from hand with a single consumer camera. The point clouds were metrically scaled by detecting scale targets of known size in the images. The grape bunch volume, bunch weight and number of berries were then extracted using empirical correction factors, convex hull, as well as meshing and CAD techniques. They reached correlation factors of >75% compared to manually-collected reference data. Considering the potential for high throughput data acquisition, the necessity to attach targets to the grapevines is not feasible. Furthermore, an automatic high throughput system would not to be able to optimize the camera positions towards individual grape bunches.

In accordance with the points made before, we aim to reach three main goals in our research:

(1) Acquisition of as much morphological data of multiple vine rows as possible, in a short period of time (high-throughput) from a moving vehicle.
(2) Automatic classification of the collected data into grape bunches and canopy.
(3) Automatic quantification of the points classified as belonging to grape bunches to derive the yield parameters berry diameter, as well as the number of berries and grape bunches.

In our initial approach, geotagged images are collected from a moving sensor platform at three different heights to reconstruct a colored 3D point cloud of whole vine rows using a commercial multi-view-stereo software. Automatic data interpretation are achieved by applying a supervised classification algorithm, which differentiates between points in the point cloud belonging either to grape bunches or the canopy by their geometry and color features. The interpreted 3D data are then quantified by applying off-the-shelf connected components algorithms for grape bunch quantification, as well as a self-written berry detection algorithm referred to as *findBerries*, which counts berries and determines their size directly from the 3D data. We demonstrate the phenotyping pipeline on two different measuring scenarios of different complexity. For the evaluation of our concept, we looked at five meters of a traditional vertical shoot-positioned (VSP) trained trellis system and a semi-minimal pruned hedge (SMPH). The accuracy of the pipeline for the extraction of the yield parameters is then evaluated using reference data.

## 2. Materials and Methods

**Figure 1:**
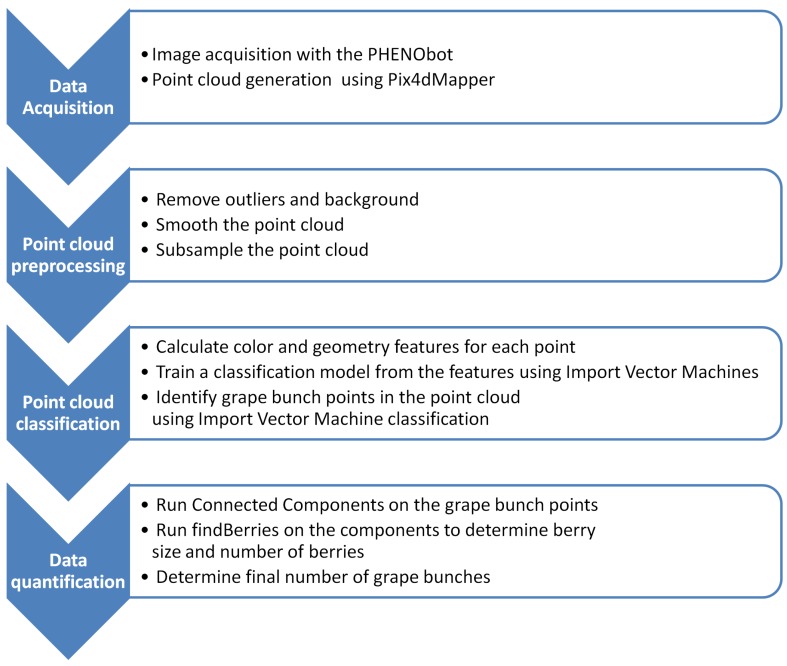
The workflow of the pipeline.

This section covers the three basic steps necessary to derive the yield parameters and describes the acquisition of reference data. Firstly, the data acquisition method in the field, as well as data post-processing and preparation are covered. Secondly, the features to describe each point in the point cloud and the classification method utilized to automatically differentiate the data are described. The third section contains the quantification methods used to derive the yield parameters. Figure 1 depicts the workflow of the pipeline.

### 2.1. Plant Material

**Figure 2:**
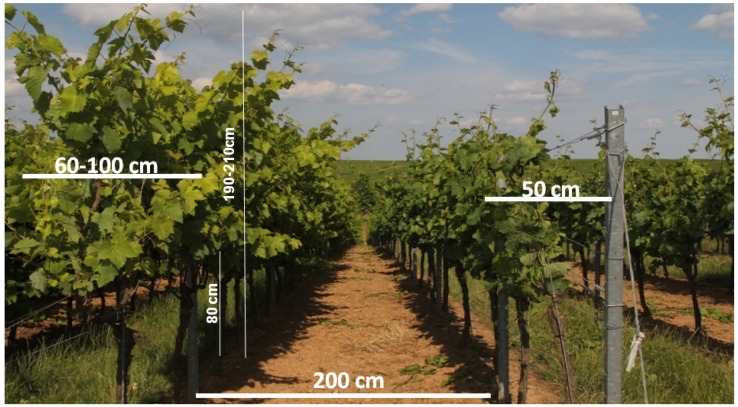
(**Left**) A grapevine row trained as a semi-minimal-pruned-hedge (SMPH) system; (**Right**) A grapevine row trained as a traditional vertical shoot positioned (VSP) trellis.

**Figure 3:**
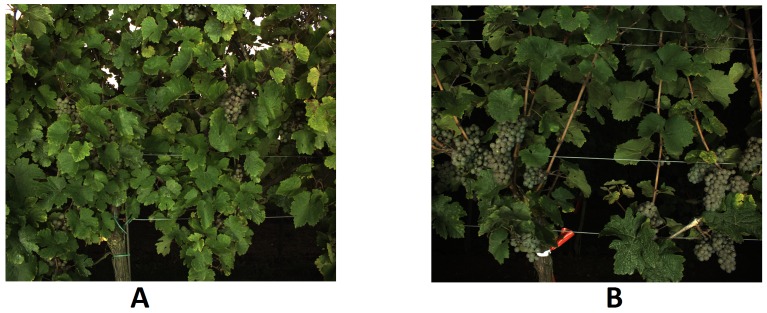
(**A**) A grapevine row trained as SMPH; (**B**) A grapevine row trained as a VSP trellis.

Two rows of Riesling, each with 25 vines, have been used for the setup of the phenotyping pipeline. Vines are planted at the experimental vineyards of Geilweilerhof located at Siebeldingen, Germany (Lat 49°13${}^{\prime }$2.892${}^{\prime \prime }$, Lon 8°2${}^{\prime }$48.408${}^{\prime \prime }$). Inter-row distance was 2 m, and grapevine spacing was 1 m. Rows were planted in a north-south direction. Vines have been trained in two different ways to demonstrate the phenotyping pipeline on two different measuring scenarios with different complexities. One row was trained in the traditional vertical shoot positioned (VSP) trellis system (see Figure 2 and Figure 3). In this scenario, the canopy density is rather low with a canopy width of approximately 50 cm and a grape bunch zone of roughly 50 cm located at the lower part of the grapevine canopy. The second row contained semi-minimal-pruned-hedge (SMPH) trained vines. These are more vigorous with a higher canopy density and width (60–100 cm). In this training system, grape bunches are allocated throughout the whole canopy height with smaller bunches and berries compared to the VSP system.

### 2.2. Data Acquisition

One of the main goals of this research is high-throughput data acquisition where near-to-complete morphological data of multiple whole vine rows is gathered in a short period of time. The sensor platform thus needs to fulfil the following conditions:

- Utilization of non-invasive sensors
- Near-to-complete 3D data from multiple perspectives to reduce the effect of occlusion
- Whole canopy height coverage
- Automatic data acquisition and storage
- Fast data acquisition from a moving platform
- Robust and largely weather-independent platform for high frequency screening
- A high level of detail for accurate measurements

**Figure 4:**
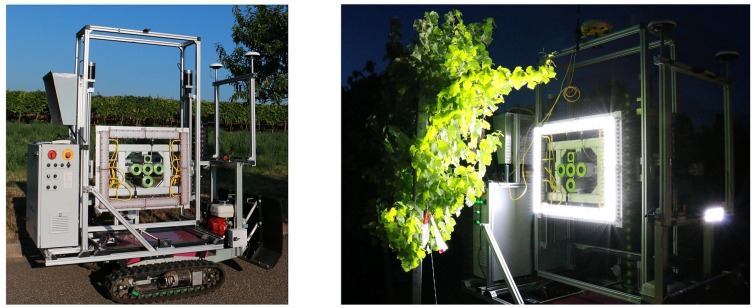
The PHENObot. RTK-GPS is attached at the top. A five camera system is attached on a camera movement frame to adjust the camera-canopy distance and camera height. The RGB camera in the middle of the camera system was used for our purposes. Around the camera system, the lighting unit is situated in a rectangular frame.

Systems built around multi-view-stereo approaches meet these requirements. We used the PHENObot [11] to test the general applicability of our concept as the PHENObot already contains most of the features relevant to our research. It was originally developed for the automatic acquisition of georeferenced single images of vine stocks for berry quantification. Figure 4 shows the PHENObot in the field. Its main components relevant to our project are:

- Track-driven vehicle with low-vibration drive for sharp images
- RTK-GPS for image geotagging (Trimble^®^ SPS852, Geo Systems GmbH, Jena, Germany)
- Lightning unit for homogeneous illumination conditions
- A 5-MP RGB camera (AVT GT-2450C; objective: Schneider KMP-IR CINEGON 8 mm; 2448 × 2050 pixels)
- Fast data acquisition and storage using our own software (IggGeotagger)

**Figure 5:**
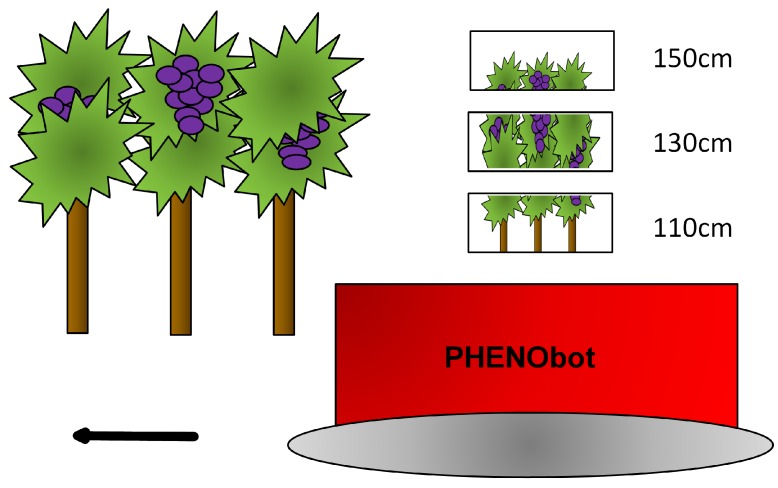
Data acquisition using the PHENObot. Please note that the three camera frames shown simultaneously only serve as an illustration for the height coverage achieved using three separate camera heights.

Please refer to [11] for a detailed description of the PHENObot and the data acquisition software. Data acquisition was done after sunset using the lighting unit to achieve homogeneous lighting conditions. The PHENObot moved between the grapevine rows with the camera looking to the right of the path direction. The distance of the camera to the grapevines was 50–75 cm. Please see Figure 5 for an illustration of the scenario.

Image acquisition was done at night to guarantee homogeneous illumination conditions. Driving speed was about 0.3 km/h. Initial camera height above the ground was at 110 cm. An image was taken approximately every 15 cm along the path while moving through the grapevine row by manually pressing a button by which the lightning unit was triggered and the GPS position automatically written in the images’ Exif (Exchangeable Image File Format) file header. Considering the driving speed, the baseline of 15 cm ensured an image overlap of 80%–90%, which is necessary for high detail and accurate point clouds. The high screening rate reduces the effect of occlusion as points of a grape bunch are seen from multiple views. In this way, the whole row was screened. To reduce the effect of occlusion and as an image taken from a height of 110 cm did not capture the full height of the grapevines, the procedure was repeated at camera heights of 130 cm and 150 cm driving along the same path as before.

### 2.3. Point Cloud Generation

A colored 3D point cloud of the whole grapevine row was reconstructed using the commercial MVS software Pix4DMapper (Pix4D SA, 1015 Lausanne, Switzerland). Pix4DMapper has already proven its user friendliness and applicability to 3D phenotyping tasks in [18]. For the reconstruction, all images were used with full resolution. The GPS positions of the images are used as approximate positions for the internal bundle adjustment of Pix4DMapper.

The advantages of geotagged images lie in a reduced processing time and the metric scaling of the finished point cloud. No scale deduction from known objects in the images [11,20,23] is needed. Camera calibration is done automatically without the need for user intervention. Approximate values for the camera parameters are derived from the EXIF data of the images. The approximate GPS positions of all images are corrected and later used for findBerries. A point cloud contains about 100 million colored 3D points and uses about 1.5 GB in memory using the ASCII encoded PLY (Polygon File Format) format. The point cloud is automatically saved into separate PLY-files. Each file contains approximately 3–5.5 m of the grapevine row in length.

### 2.4. Point Cloud Preprocessing

**Figure 6:**
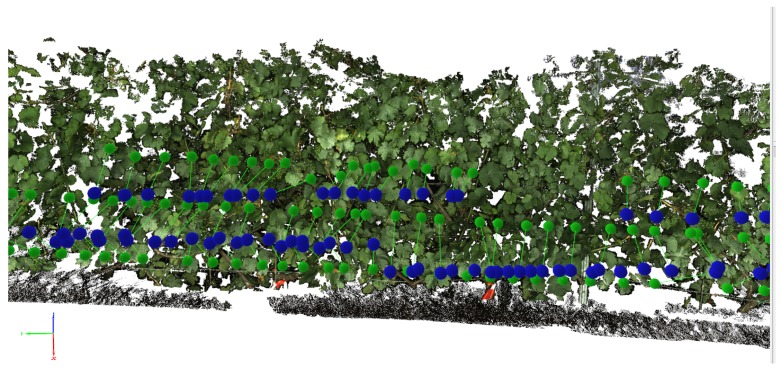
Part of the reconstructed SMPH grapevine row (25 m). The dark background is clearly visible. Blue spheres indicate initial GPS positions of the cameras. Green spheres indicate final camera positions corrected during bundle adjustment.

Before data interpretation, the point cloud has to be prepared. Each PLY file is processed separately, containing about 10–15 million points. The point cloud contains dark, non-existent points on object borders resulting from mixed pixels between the black background and the object. It further contains outliers and noisy surfaces (Figure 6). All of these factors corrupt the geometry of the point cloud. First, the night black background is removed using simple color thresholding within a MATLAB 2009b script (The MathWorks Inc., Natick, MA, USA). In order to do this, the color of the point cloud is transformed into the HSV color space (hue-saturation-value). The HSV color space is a cylindrical-coordinate representation of a color based on the three-axis HSV. Hue contains the color information; saturation encodes the color depth; and value the brightness. In phenotyping and image segmentation, it is frequently used [24,25,26]. RGB space depicts color as a percentual combination of all three channels, while the color information in HSV is expressed with a single H-value. All points below an empirically-deduced value of V for background points are removed. It significantly sharpens the contours of objects and removes dark regions in the background, which exhibit a reduced geometric accuracy. HSV filtering removes points based on their brightness. Though this is meant to remove the black background, some grape bunch points lying inside shadows may be removed, as well. Approaches to address this issue are presented in the Discussion.

All further preparations are done using the open-source software CloudCompare [27]. Outliers are removed using the statistical outlier removal (SOR) filter. A point’s distance to a user-defined number of neighbors is measured and compared with the average point distance. If the point lies outside the average distance plus an additional sigma multiplied with a user-defined factor, it is rejected. The cleaned point cloud is then smoothed using the Point Cloud Library (PCL) plugin Moving-Least-Squares (MLS). MLS is an approximation method where points defining a surface are smoothed locally by utilizing only the points in a user-defined neighborhood. A local plane of defined size is approximated from neighboring points within a radius around a source point. A polynomial is then used to approximate the distance of the neighboring points to the local plane. This procedure is repeated for every point in the cloud point, resulting in a smooth surface. Please refer to [28] for details on the method. The smoothed point cloud is then subsampled to a minimum point-to-point distance of 1 mm to reduce the number of points to a workable amount (please see Section 4). This point-to-point distance has proven to be useful in the context of automatic grapevine classification [29]. Around 1–3 million points per 3.5–5 m remain.

### 2.5. Point Cloud Classification

Manually segmenting the point clouds into semantic plant components is time consuming and prone to human errors. Automatic data interpretation is thus needed to generate precise and objective phenotypic data while saving time and working resources. Point cloud classification involves two steps. First, all points are classified into either belonging to a grape bunch or the canopy using the supervised machine learning algorithm import vector machines (IVM). In a second stept, the classification results are then further improved using a graph cut energy minimization approach.

#### 2.5.1. Surface Feature Histograms

Any classification algorithm needs so-called features to differentiate the data. A 3D point initially has three features describing it, namely its coordinates in *X*, *Y* and *Z*. These are not sufficient to differentiate between complex semantic objects. We therefore use a set of local geometry and color features to describe each point. Geometry features are dependent on accurate geometries and are sensitive to noise in the data. Further, geometries may be obscured because objects overlap or because they lie far away from the camera. Classification with color features, on the other hand, depends on the lighting conditions and current color expression of the objects. Plant organs are especially variable in their color appearance, e.g., due to ripeness or health status or fungicide application [9]. Our idea then is to make the features of a class more robust by using both feature types for classification. All features are calculated using MATLAB 2009b.

**Figure 7:**
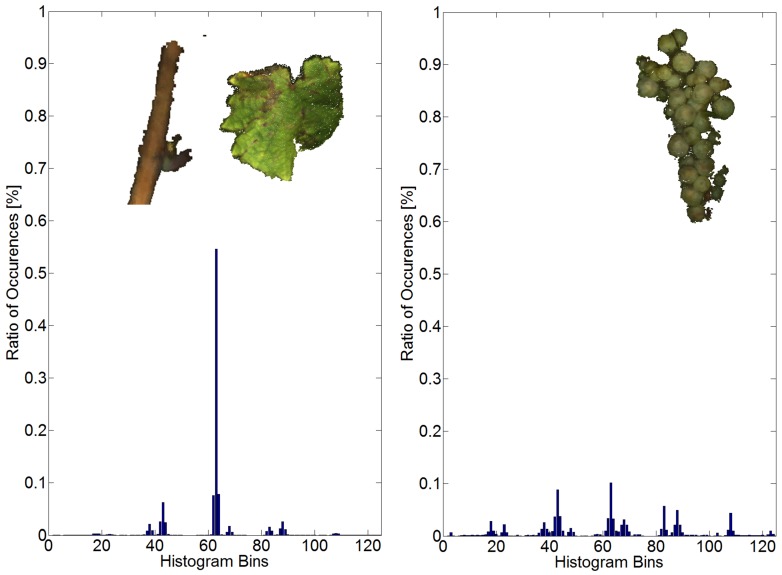
(**Left**) Typical surface feature histogram (SFH) for the canopy; (**Right**) Typical SFH for grape bunches.

Surface feature histograms (SFH) describe the geometry of a surface around a point in a local neighborhood. The general concept was first introduced in [30] in a robotics context and later adapted to plant phenotyping in [29]. We chose the latter adaption, as the SFH has proven its usefulness for classification of several plants, including wheat ears, barley and grapevines [17,29]. The local surface around a point is described by summarizing occurrences of angular values into a histogram with 125 bins. The calculation of an SFH thus gives 125 geometric features per point. The angular values are calculated using the points of the local surface and bear the advantage of being pose invariant. Different surface geometries like planes or spheres result in distinctive histograms, which can be used by classification methods to distinguish the data. Figure 7 illustrates the typical SFH for the two classes, canopy and grape bunches.

Relevant parameters for the calculation of the SFH are the radius *r_N_* for the point normal calculation and the radius *r_H_* for the calculation of the histogram. Besides the surface geometry, they alone determine the characteristics of the SFH of the point. Different radii produce different SFH characteristics. It is critical to choose fitting radii so that distinctive SFHs for each class are calculated. A grapevine mainly consists of leaves, stems, small branches and grape bunches. While leaves and thin branches can be considered to be locally planar, the surface of larger stems and grape bunches can be considered to be locally spherical or cylindrical. It is thus crucial to chose values for *r_N_* and *r_H_* that detect the planarity of leafs and the spherical geometry of grape bunches, but at the same time are able to distinguish the latter from larger stems. The smallest surface variations occur on the grape bunches where the berries form “ridges” and the space between berries forms “valleys”. The slope of a berry covers its radius. The radius is around 6 mm as obtained from manual measurements. We thus chose a value of 3 mm for *r_N_*, which captures the berries’ slope. For *r_H_*, we chose a value of 9 mm, which captures the typical “valley-ridge” geometry for one and a half berries. This geometry is not typical for larger stems of the cylindrical form and still small enough for leaves to be regarded as locally planar. Using several other radii combinations did not result in SFHs as distinctive as seen in Figure 7.

#### 2.5.2. HSV Color Features

Besides the point cloud, Pix4DMapper gives RGB color information for each 3D point. It was shown that HSV is superior to the RGB space for image segmentation tasks [24,31]. The RGB information of each point is therefore transformed into the HSV space.

The three HSV features serve as additional color features to the 125 geometric features, obtaining 128 features for each point.

#### 2.5.3. Supervised Classification Using Import Vector Machines

**Figure 8:**
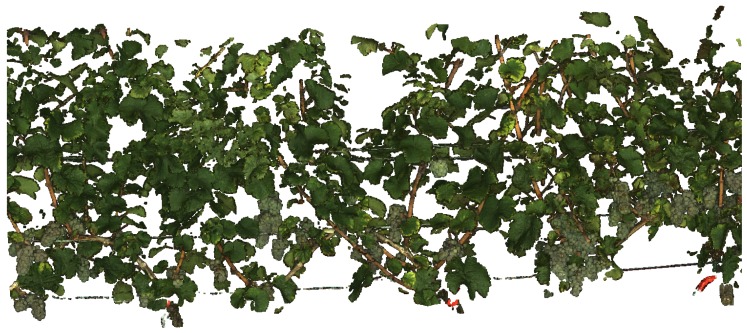
Original VSP trellis point cloud.

We use the supervised classification method incremental import vector machines (I^2^VM) to classify our data [32,33]. In supervised methods, the data representing a class are chosen by an analyst based on his/her experience to include all possible class morphologies. The underlying assumption is that the features of a class remain relatively constant throughout all class instances, enabling definite class definitions. In unsupervised classification methods, classes are built by calculating features for the data first and defining classes afterwards based on some measurement of feature similarity (e.g., in K-means clustering) [34]. While in this way, unknown classes can be detected, the disadvantage of using unsupervised methods within plant phenotyping is the variability of plants. An experienced analyst knows the class morphology and has control over which data to include in the class definition. The unsupervised method, on the other hand, may create classes that do not correspond to actual plant components or mix different plant components into one class because their features are similar. Figure 8 depicts a part of the original point cloud.

Import vector machines are supervised kernel-based classifiers. It has been shown that they are competitive with or superior [32,35] to other machine learning methods, like the popular support vector machines (SVM) [36]. The IVM requires training data in the form of features that describe each data point to generate a discriminative classification model. As training data, points representative of the classes grape bunches and canopy are manually chosen from the point clouds using Geomagic Studio 12 (Raindrop Geomagic Inc., Morrisville, NC, USA). Attention focused on including all possible geometries and colors a class can possess to generate a comprehensive classification model. The number of training points for each class should be balanced to avoid overmatching. About 600 points of each class were used as training data. Each class is assigned a unique class label *i* ∈ *n*, where *n* is the number of classes, two in this case. For every point of the training data, the SFH and HSV features are calculated, forming a 128-dimensional feature vector. Using these features and the respective class label for each point, the classification model is built by the IVM. The features form a so-called feature space, in which a so-called hyperplane is built as a border to separate points belonging to different classes. This hyperplane is the classification model. For the classification of the five meter-long point clouds of the SMPH and VSP trellis rows, the SFH and HSV features are calculated for every point. The output of IVMs is probabilistic, i.e., the probabilities of class membership are estimated. Using the same classification model for both rows, the IVM estimates the probabilities of class membership *P_x_* = [*p*_1_, ..., *p_n_*] for each point *x*, whereby *n* = #classes. *P_x_* is a real estimate of the posterior [10], while SVMs on the other hand either give direct class assignments or only pseudo-probabilities [37]. A point is then assigned to the class with the highest probability. The classification using the IVM is referred to as initial classification.

**Figure 9:**
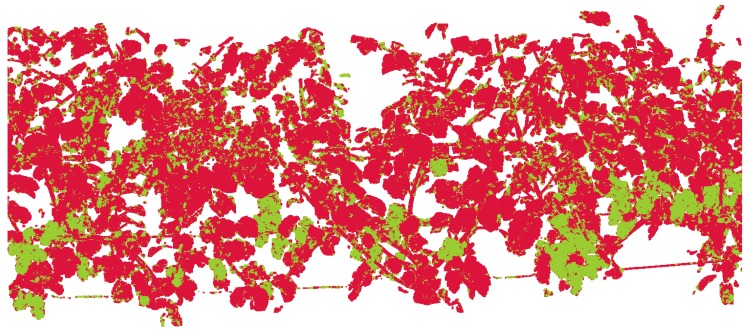
Initially classified point cloud. Red points belong to the canopy and green points to the grape bunch class. False classifications are recognizable, especially where objects lie close together. Point regions belonging to the grape bunch class contain points falsely classified as canopy and vice versa.

Figure 9 shows the initial classification results for the part shown in Figure 8.

#### 2.5.4. Label Smoothing (Handling Classification Noise)

Each point is classified independently without incorporating spatial information about the assigned class label of its neighboring points. Classification noise is defined as points of a diverging class inside mostly homogeneous point regions of another class (Figure 9). We address this issue by spatially smoothing the class labels of the initial classification. The open-source MATLAB software GCO (GCoptimization) [38,39,40] interprets the 3D point as nodes in a graph cut energy minimization approach. The nodes of the graph are connected via the neighborhood relations between points within a user-defined radius. We use the class membership probabilities *P_x_* estimated by the IVM classification as the energy function to be minimized by GCO .

**Figure 10:**
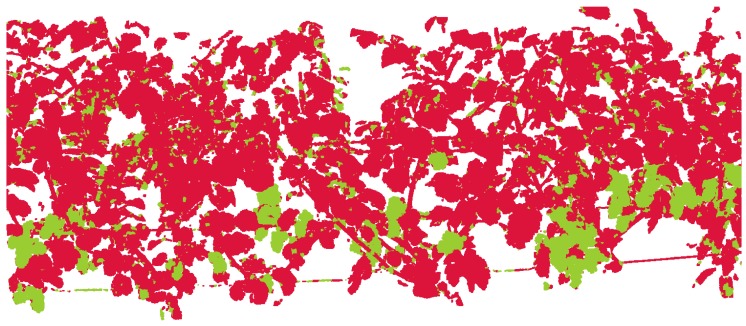
Classified point cloud after label smoothing. Former heterogeneous regions are now homogeneous. Small regions of misclassified points are corrected. Branches lying in between grape bunches are classified correctly.

Each node then contains the sum of the class membership probabilities *P_x_* and a user-defined penalization term for diverging labels. GCO minimizes this energy and eventually assigns new class labels to the points. This method significantly enhances the results of the initial classification, as shown in Figure 10, and is referred to as label smoothing.

### 2.6. Data Quantification

Yield parameters we are considering in our phenotyping pipeline are the number of grape bunches and berries and the berry diameter. After classification, all points belonging to a grape bunch are first segmented into spatially-distinct point regions. These serve as a basis for the quantification task, in which the number of grape bunches and berries, as well as the berries’ size are determined. We first describe our algorithm findBerries for berry detection and approximation. Afterwards, the yield parameter estimation using points belonging to the grape bunch class is explained in detail.

#### 2.6.1. FindBerries

The following passage describes our algorithm to count berries in a grape bunch and measure their radius. We know that grape bunches are composed of berries exhibiting a spherical geometry. The algorithm is therefore built on the assumption that the point cloud in which berries are searched for is a grape bunch where no other geometries are expected.

**Figure 11:**
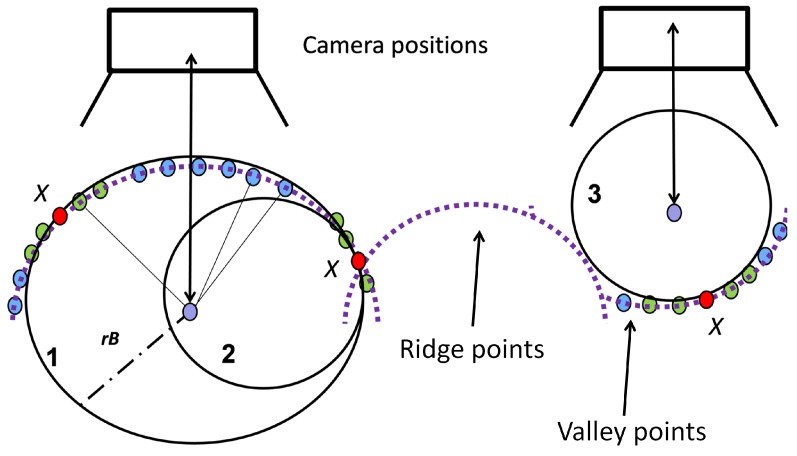
Scenario for berry quantification. Purple lines illustrate points of a grape bunch viewed in profile. Red circles depict the source point *X*, green circles the points used for the sphere approximation and blue circles the points within the sphere radius *r_B_*. Spheres 1–3 are spheres with a valid sphere radius and enough supporting points. They depict scenarios in three different evaluation stages. Sphere 1 is accepted; Sphere 2 is rejected because it overlaps with Sphere 1, and its support is smaller than that of Sphere 1. Sphere 3 is rejected because it lies in the space between berries, in a “valley”.

A source point *X* from a grape bunch point cloud is chosen randomly. Points within a user-defined spherical neighborhood around the source point are used to approximate a sphere. Now, a hierarchical evaluation to assess if the sphere approximation corresponds to a berry is performed. Figure 11 shows a grape bunch from a side view. Three different evaluation stages of berry quantification are depicted.

(1) Radius *r_B_*: Is the sphere radius typical for berries in their momentary ripeness state? Reject radii lying outside the expected value range.
(2) Support: How many points encompassed by the sphere support the sphere approximation? Calculate the distance of these points to the sphere’s middle point and compare them with the sphere radius. Points with a distance diverging by more than 10% of the sphere radius are counted as outliers. Calculate the outlier-inlier ratio and apply threshold filtering.
(3) Position: Does the sphere approximate the space between berries (“valley”) or a berry (“ridge”) (Sphere 3, Figure 11)? Detect spheres lying in the space between berries using the camera positions given by Pix4DMapper. A straight line from the sphere middle point to the nearest camera position is created. If enough points lie on the line, the sphere represents a berry; otherwise, the sphere is located in the space between berries and rejected.
(4) Sphere overlap: Spheres overlapping each other with a ratio exceeding a defined threshold are tested for the size of their support (Spheres 1 and 2). The sphere with the better support is kept as a valid berry approximation.

If the berry approximation is considered valid, all support points are removed from the remaining point cloud. The next source point *X* is chosen and the evaluation repeated. The algorithm stops when no further valid approximations after 2 · #_remaining points_ iterations were found.

#### 2.6.2. Quantification of Grape Bunches and Berries

Points classified as belonging to the grape bunch class do not give information on the number of grape bunches existing in the grapevine row, merely that they are of that class. Further, misclassified regions actually belonging to the canopy class are still present after label smoothing with GCO. Therefore, all grape points are divided into individual sub-point clouds using the algorithm Connected Components available in CloudCompare. Points lying closer together than a user-defined distance threshold form a point region, a so-called component. First, a distance threshold of about 5 mm is used. Components whose number of points lies under a user-specified threshold are deleted. The threshold is chosen based on a priori knowledge about the minimum number of points of which a grape bunch typically consists. For VSP trellis, the minimum number is 400 points, for SMPH 300 points. In this way, the small regions of misclassified points are deleted. Actual grape bunches or grape bunch parts may be deleted this way. Deletion of actual grape bunches is accounted for in the recall of the final results. Components exceeding a certain number of points are then subdivided using Connected Components a second time, as a high point number usually indicates the fusion of several grape bunches into one component. For the VSP trellis, the number of points lies at 5000 points, for SMPH at 3000 points. A smaller distance threshold of about 1 mm is then used to separate point regions close together.

As intermediate result, the remaining components are treated as potential individual grape bunches. Each component is then processed individually. In each component, berries are searched for using findBerries. Components where less than three berries are found are rejected. The number of remaining components constitutes the final grape bunch yield parameter. The sum of the number of berries found in each component and their respective size constitute the final yield parameter result for berries.

### 2.7. Evaluation Metrics

Accuracy assessment is done by calculating three error metrics. We use the RMSE (root-mean-square-error) to evaluate the deviation of an actual measurement from a reference measurement. For the classification and final yield parameter estimation, the percentual metrics recall and precision are used. They are calculated class-wise, e.g., for the grape bunch class, and require reference data. Using the reference data, the numbers of true positives, false positives and false negatives are counted. The recall in the example of grape bunches answers the following question: How many points belonging to the grape bunch class really present in the vineyard were found, e.g., through classification? Precision then is the complementary metric to the recall and answers the following question: How many points predicted to be belonging to the grape bunch class really are grape bunches? A recall of, e.g., 80% then tells us that the points predicted, e.g., as a grape bunch constitute 80% of the true grape bunch points present in the point cloud. A precision of, e.g., 100% then tells us that each point classified as a grape bunch point really is a grape bunch point.

### 2.8. Reference Data

For the evaluation of the classification with IVM, reference data are needed. Points in the five meter-long point clouds of the VSP trellis and SMPH grapevines belonging to grape bunches and the canopy were manually divided using Geomagic and manually labeled using MATLAB. In this way, the class of each point in the five meter-long point clouds was determined and used as the reference data for the calculation of recall and precision.

For the evaluation of findBerries, an artificial grape bunch was used. The high accuracy close-up triangulation line scanner Perceptron v5 (Perceptron Scan Works V5, Perceptron Inc., Plymouth, MI, USA) combined with an articulated measuring arm (Romer Infinite 2.0 (1.4 m), Hexagon Metrology Services Ltd., London, UK) was used to reconstruct the artificial grape bunch with a point-to-point resolution of 14 µm and an accuracy of 45 µm. The point cloud serves as highly accurate reference data. Berries in the point cloud were counted manually. For size reference, the diameter of 20 berries was measured with a caliper, whereby the diameter varied between 15 and 20 mm. The RMSE for the berry diameter is then calculated using the diameter estimation derived from findBerries and the manual measurements.

Reference data for the evaluation of the number of grape bunches and berries is collected visually by counting occurrences in the images used to reconstruct the point cloud. Reference data for the number of grape bunches is collected by the meter on five meters of VSP trellis and SMPH grapevines. Reference data for the number of berries are generated from 10 individual grape bunches of VSP trellis and SMPH grapevines. We use recall and precision to evaluate the results derived from the whole pipeline. The estimated berry diameter is compared to 100 reference measurements collected in the VSP and SMPH rows using a caliper.

Images from the VSP trellis row are available at the Open Agrar Repository [41] for research purposes.

## 3. Results

In this section, the results from data acquisition and point cloud preparation, point cloud interpretation and yield parameter estimation are presented.

### 3.1. Data Acquisition and 3D Reconstruction

**Figure 12:**
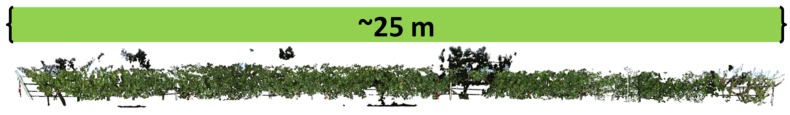
The point cloud of the whole 25 m-long VSP trellis row.

Approximately, 700 images per row (about 10 GB) were captured in total, depicting the whole length and height of the row. The screening of the whole grapevine row in the three camera heights took about 15 min. Point cloud reconstruction took about eight hours using a PC with 12 GB memory, producing a point cloud with approximately 100 million points distributed into separate PLY files. The grapevine row of 25 m was reconstructed in its entire length (Figure 12) and prepared as explained in Section 2.4.

**Figure 13:**
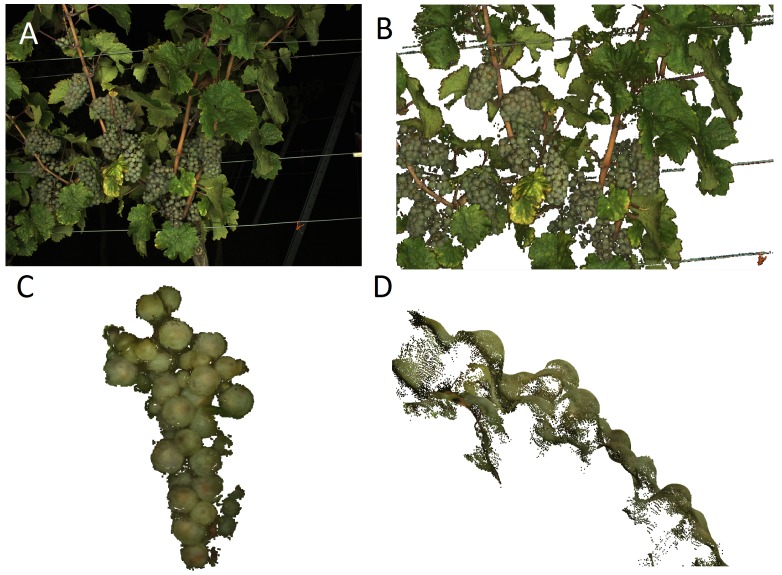
(**A**) Original RGB image of a VSP trellis row; (**B**) Cleaned and smoothed point cloud of the same scene; (**C**) Front view of a grape bunch; (**D**) Side view of the same grape bunch. Elevations of single berries are clearly distinguishable, forming a “valley-ridge” geometry (**B**–**D** not yet subsampled for visualization purposes).

Figure 13 shows parts of the final point cloud. All object contours are sharpened through background removal. Figure 13C,D illustrates the level of detail and smoothness of the point cloud. Individual berries are clearly distinguishable, forming a smooth “valley-ridge” geometry. Sides of berries are reconstructed through the MVS approach, which makes sphere approximation more reliable through point redundancy. Grape bunches deeper inside the canopy were reconstructed, as well, which shows that the MVS approach is effective in compensating for occlusion.

### 3.2. Point Cloud Classification

This section covers the classification results. As we are only interested in the grape bunch class, results for the canopy will be neglected. We give values for recall and precision for VSP trellis and SMPH grapevine rows. Classification of about 1.5 million points takes about five minutes. The result from the initial classification is drastically improved through GCO. Table 1 and Table 2 show the recall and precision results classification with IVM and after label smoothing with GCO for a VSP trellis and an SMPH grapevine row.

The initial classification for the VSP trellis row gives a recall of 86.6% and a precision of 38.8%. Label smoothing enhances the recall to 94% and the precision to 62.8%.

The initial classification for the SMPH row gives a recall of 81.2% and a precision of 23.2%. Label smoothing enhances the recall to 88.7% and the precision to 38.4%.

For completeness, results for the classification of the same VSP trellis row using the SFH and HSV features individually were produced. Table 3 shows the results after label smoothing. The results indicate that combining geometry and color features has a beneficial effect on the recall, but lessens the precision. Our focus lies on extracting near to complete data, which necessitates the recall to be high. We therefore prioritize a high recall in this processing step, as remaining classification noise (false positives) is reduced later on through utilizing Connected Components during the estimation of the yield parameters.

### 3.3. Yield Parameter Estimation

This section deals with the evaluation of findBerries and the final yield parameter results regarding berry diameter, as well as the number of berries and grape bunches. Recall and precision for all yield parameters are calculated using the reference data.

#### 3.3.1. Evaluation of FindBerries

**Figure 14:**
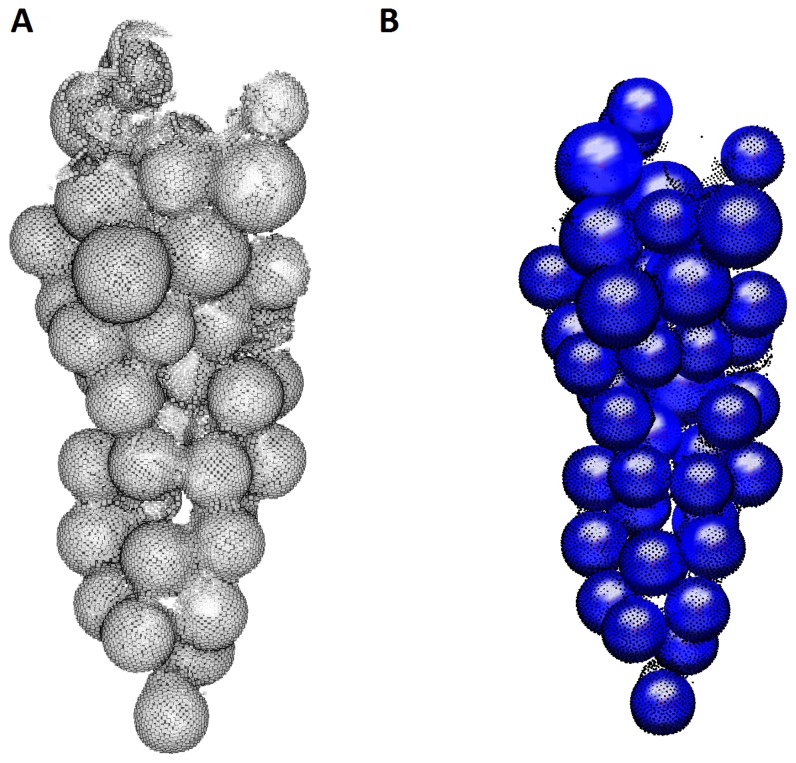
(**A**) The grape bunch scanned with the Perceptron line scanner; (**B**) The spheres found by findBerries. Partially-reconstructed berries suffice for correct sphere approximation.

The high detail laser point cloud of the artificial grape bunch serves as reference data. All artificial berries were found by the algorithm. All berries found by findBerries correspond to real berries. Comparing the manually-measured diameter to the algorithm result gives an RMSE of 0.5 mm. Figure 14 shows the grape bunch point cloud and the found berries. It can be concluded that findBerries works very well if the 3D data represent the true geometry accurately.

**Figure 15:**
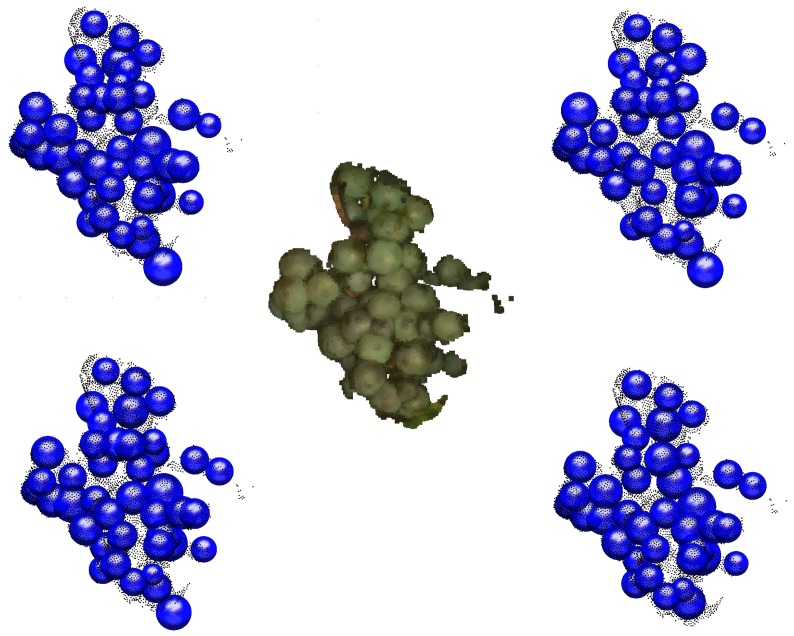
The original grape bunch point cloud is seen in the middle. In the corners, exemplary results of four runs of findBerries are depicted.

The algorithm findBerries is a randomized approach, as the source point for sphere approximation is randomly chosen. It was thus further tested for repeatability on one example grape bunch from the field data. For this purpose, findBerries was run 20 times on the example grape bunch, using the same parameters each time. It showed a standard deviation of 1.5 berries. A visual inspection of four out of the 20 different plots of the same grape bunch demonstrates that the berry detection is stable (Figure 15). Most berries are found reliably, with some berries missing in some cases. The issue of missing berries is addressed in the Discussion.

The results indicate that findBerries is a suitable tool for berry detection and size estimation. They also show that the success of findBerries is dependent on the quality of the 3D data.

#### 3.3.2. Grape Bunch Yield Parameter

**Figure 16:**
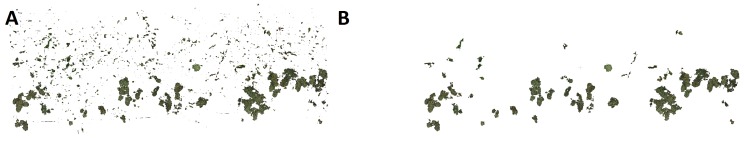
(**A**) Points classified as grape bunch class. Classification noise can be noticed as small, unstructured point regions; (**B**) The component size discrimination clears most of the misclassified point regions while keeping the actual grape bunches.

The Grape bunch yield parameter is derived using the method outlined in Section 2.6. Figure 16 shows the points of the grape bunch class before (Figure 16A) and after (Figure 16B) connected components were applied.

**Figure 17:**
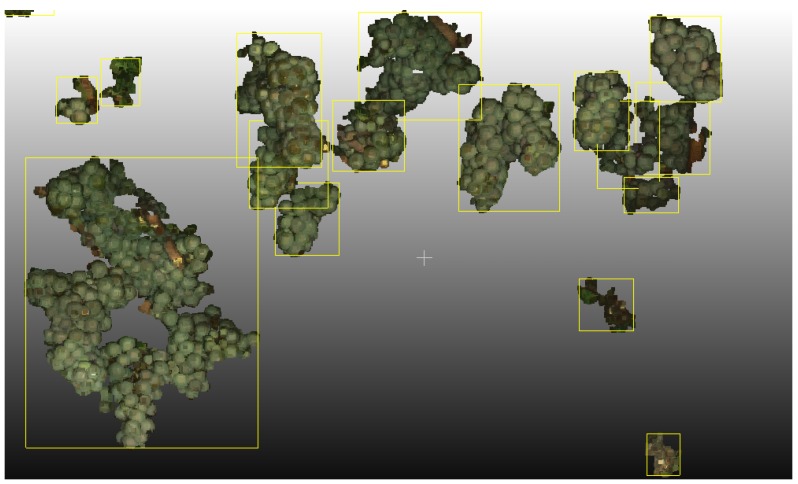
Components detected, indicated by a yellow bounding box.

True grape bunches not reconstructed by Pix4DMapper or deleted during yield parameter estimation count as false negatives. A component consisting of several grape bunches, which cannot be differentiated spatially because they are close together, counts as one grape bunch only. For example, a component could consist of five true grape bunches, but is only counted as one grape bunch. This situation would produce four false negatives. Components, which are not actual grape bunches, count as false positives. One true grape bunch divided into, e.g., two components produces one true positive and one false positive. Figure 17 displays several components, indicated by a yellow bounding box. The large single component on the left consists of several true grape bunches and is therefore treated as one grape bunch only. In the right bottom region, components not corresponding to grape bunches can be seen.

Table 4 shows the recall and precision for the grape bunch yield parameter of a VSP trellis row for the 5 m × 1 m sections. Here, $\bar{Meter}$ denotes the mean value, while *σ* denotes the standard deviation. The mean for the recall is slightly higher than the precision, while its standard deviation is higher with 16% compared to 6%.

In total, there were 79 grape bunches visible in the images of the VSP trellis row. Out of these, nine grape bunches were missed because they were not reconstructed and therefore not present in the point cloud. No grape bunches were lost during classification. Another 10 were not detected by the pipeline during Connected Components or findBerries. Six of these were fused with other grape bunches and therefore not counted as individual grape bunches.

Table 5 shows the recall and precision for the grape bunch yield parameter of the SMPH row. Here, the mean for the recall is lower than the precision. The standard deviation of the recall is lower with 7% than for the precision with 9%.

In total, there were 61 grape bunches visible in the images of the SMPH row. From these, 14 were missed because they were not reconstructed and therefore not present in the point cloud. No grape bunches were lost during classification. Only one grape bunch was not detected by the pipeline during Connected Components or findBerries.

#### 3.3.3. Number of Berries and Berry Size

**Figure 18:**
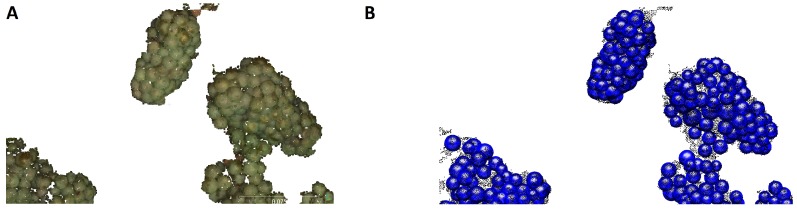
(**A**) Original points classified as grape bunch class; (**B**) Berries detected on several components. Almost all berries present in the point cloud are found, with some missing in areas where the spherical shape was not reconstructed properly.

Berries are detected using findBerries on each component. The detected berries are checked if they correspond to the respective true berries visible in the images. Detected berries not corresponding to true berries are counted as false positives. Berries not detected count as false negatives. These include true berries, which are not present in the point cloud. For berry diameter evaluation, the histogram of the approximated berry diameter is compared to the histogram of the reference data since no direct berry-to-berry reference was measured. Figure 18 shows recall and precision results for berry detection on 10 grape bunches reconstructed and classified under field conditions.

Table 6 shows the recall and precision for berry detection on 10 large and medium-sized grape bunches of the VSP trellis row. Recall is 77.6%, while precision is higher with 97.8%. The standard deviation is 13.2, resp. 2.6%.

**Figure 19:**
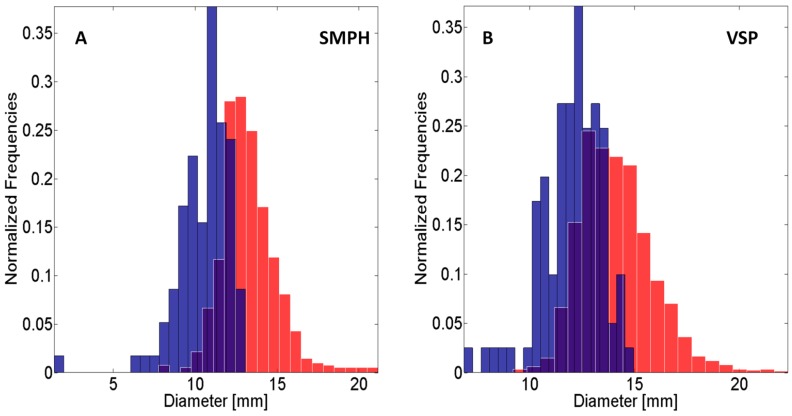
(**A**) Normalized histograms for the berry diameter of the SMPH row; (**B**) Normalized histograms for the berry diameter of the VSP trellis row. **Red**: Estimated with findBerries. **Blue**: Measured manually in the field. **Purple**: Area of overlap between both histograms.

Table 7 shows the recall and precision for berry detection on 10 large and medium-sized grape bunches of the SMPH row. Recall is 77.2%, while precision is higher with 97.6%. The standard deviation is 10.8, resp. 3.3%. Figure 19 shows the histograms for the berry diameter. Figure 19A shows the results for the SMPH row and Figure 19B for the VSP trellis row.

Table 8 shows the final yield parameter results for five meters of grapevines for the VSP trellis and SMPH method. For the VSP trellis row, 1577 berries were detected. The estimated mean diameter is 13.5 mm, while the measured mean berry diameter is 12.1 mm. For the SMPH row, 629 berries were detected, with an estimated mean diameter of 12.7 mm and a measured mean diameter of 10.7 mm.

## 4. Discussion

Each separate step of the pipeline will be discussed regarding potential for improvement in a faster, more sophisticated and automated pipeline.

### 4.1. Sensor Platform and Data Acquisition

The MVS approach equipped with GPS has proven to be an effective data acquisition method from a moving platform. As only one side of the grapevines is reconstructed, empirical correction factors have to be determined to account for the non-visible side. The usage of GPS makes the application of artificial scale targets in the grapevines obsolete, saving working time. It also enables automated management of the results in a geoinformation system. Collecting data at night using an external lightning unit has proven to be important for homogeneous lightning conditions, but is impractical as a daily work routine. Because the PHENObot is equipped with only one RGB camera, it takes three traverses to screen a row completely, which is too much for high-throughput ambitions. We are currently in the process of designing and building a new sensor platform where these problems are addressed. Vertical image overlap will be increased through a multi-camera system to reach a complete screening of the row in only one traverse of about one minute. Multiple lightning units will be used inside a darkened “chamber” to reach daytime independence and to deepen the illumination level inside the canopy. GPS will be attached for image geotagging, as shown in this phenotyping pipeline. We hope to increase the recall of grape bunches and berries with the new system.

### 4.2. Point Cloud Classification

The recall of the grape point classification is high with 89%–94% for both grapevine rows. No full grape bunches present in the point cloud were missed by the classification algorithm. The missing points do not constitute a full grape bunch, but are rather distributed among all grape bunches, so that only small grape bunch parts are missing from each grape bunch due to misclassification. It could be shown that label smoothing using GCO improves both recall and precision to a large degree. Precision is higher for the VSP trellis row compared to the precision for the SMPH row. Both precision values are lower than the recall. Small areas of misclassified points remain after label smoothing, especially for the SMPH row, probably due to the denser and more complex canopy of the grapevines, which may corrupt the geometry with overlapping objects and a higher occlusion rate. An initial comparison hints that the usage of combined SFH and HSV features seems to be beneficial to the recall, while obtaining a solid precision. Still, more detailed experiments need to be carried out to test the effectiveness of the features. It could be demonstrated that the same training data for both training systems can be used. The approach yielded good results, although both rows are of the Riesling variety, their morphologies vary drastically. For other varieties, new training datasets have to be generated to account for geometry and color differences of the plants.

### 4.3. Yield Parameter Estimation

Using a Connected Components approach proves to be an effective tool for grape bunch yield parameter estimation in a 3D scenario. Size thresholding removes most of the misclassified point regions. Neighboring grape bunches within a distance of 1–2 mm can still be distinguished. The approach is simplistic in that the only parameter is the distance. This is reflected in the standard deviation of the recall. It fails where grape bunches are situated closer together than 1–2 mm. Multiple grape bunches are then counted as one grape bunch only. One grape bunch may also be divided into several grape bunches because a branch caused a division or because some parts of the grape bunch are missing. The remaining regions of misclassified points need to be addressed, as well. Their geometry is unstructured and unpredictable (Figure 17), with a large bounding box, but a sparse point distribution. Algorithms making use of the homogeneous and denser point distribution in actual grape bunches, either of the points themselves or their berries, may solve the problem. Connected Components then could serve as an initial result.

The estimation of the number of berries reaches a high precision with a lower recall. The results for both rows correspond to the grape bunches where recall has a higher standard deviation than the precision. Recall is lower for two reasons. Parts of a grape bunch may be missing due to not being reconstructed or not being classified as grape bunches. Typically, these berries lie deeper inside the canopy and on the outer sides of the grape bunch. It is thus difficult to capture them from multiple camera views. Another factor is the imperfect spherical shape of berries reconstructed under field conditions and the random approach of findBerries. Depending on the points used for the sphere approximation, the sphere size may vary from 0.5–1 mm for the same berry. Caliper measurements exhibit the same uncertainty due to the softness of the berry or the variation of the contact points of the caliper. Points supporting the sphere approximation may then include points belonging to another berry, which are then missing for sphere approximation. Depending on image overlap and how often a point is seen in different images, the spherical berry geometry may not be reconstructed properly. Sphere fitting is not possible if the surface is too flat. We hope to enhance geometric accuracy with our new sensor system where image overlap and illumination are improved.

### 4.4. Final Yield Parameters Regarding SMPH and VSP Trellis-Trained Grapevines

The final yield parameter results for grape bunches and berries already reveals the differences between the VSP trellis and SMPH method. Fewer grape bunches and berries were found in the SMPH row than in the VSP trellis row in total. The recall for grape bunches of the SMPH row is lower than for the VSP trellis row. More grape bunches of the SMPH row were missed during reconstruction than in the VSP trellis row. This difference is probably caused by the denser and thicker canopy where the occlusion rate is higher. For the VSP trellis row, six grape bunches were missed by the pipeline because of their spatial proximity. For the SMPH row, only one grape bunch was missed by the pipeline. The grape bunches of the SMPH row resign in all canopy heights are smaller and lie farther away from each other, making their spatial differentiation with Connected Components easier. Berry diameter and berry number is decreased for the SMPH-trained system compared to VSP. The results show that the estimated diameters correspond to the manually-measured diameter up to approximately 2 mm. This difference could be caused by the geometric accuracy of the point cloud or in the valid sphere radii used for findBerries. Another explanation could be the temporal difference of 14 days between the manual measurements and the collection of the images in late September where the berries might have continued growing. The relative diameter differences between two separate raising methods are 0.9 mm for estimated, resp. 1.4 mm for manually-measured diameters. Additional experiments with direct berry-to-berry reference data have to be carried out.

### 4.5. Creating New Bottlenecks

The large amount of data to be processed in every step and the flexibility of the pipeline create new bottlenecks in phenotyping [42,43,44]. The image data size is very large, encompassing about 14 GB of data per row. Using large and fast storage media is inevitable if large areas are to be screened. Hardware enabling image saving speeds of at least two frames per second per camera is necessary if driving speed is enhanced to 1 km/h and an image overlap of at least 80% has to be ensured.

The reconstruction speed of Pix4DMapper depends on the working memory. The processing of about 700 images with 12 GB of working memory takes eight hours. Computers need to be equipped with a larger working memory to reduce the processing time to a feasible term. Currently, the pipeline is run individually for every PLY file due to the large number of 3D points. Here, a large working memory is again of utter importance to reduce processing time. The calculation of the SFH features for 1–1.5 million points takes approximately 10–15 min. The features claim about 0.7–1.4 GB of memory in MATLAB binary format. Generating the classification model takes about five minutes for about 600 training data points per class. This runtime increases with the amount of training data, but a one-time generated model can be used for all future classification task. The classification itself takes about five minutes for 1–1.5 million points. Feature calculation speed and the number of points that can be classified in one run need to be enhanced. Regarding automatization and user friendliness, the pipeline currently requires the manual selection of several files, e.g., the classification model and the usage of individual software tools. All processing steps could benefit from a consistent implementation in MATLAB, which would enable the utilization of the GPU and/or their parallelization in cloud server solutions, reducing the processing time for whole vineyard rows drastically. In effect, the whole system could reach a high throughput characteristic not only in data acquisition, but data processing, as well. All algorithms could then be fused into a user-friendly GUI where all steps are worked through automatically. The applicability of the pipeline to a variety of scenarios needs to be broadened through the generation of adapted training datasets for the classification and through customized parameters depending on the morphology of the grapevines.

## 5. Conclusions

We demonstrated a pipeline for yield parameter estimation using 3D data. Estimations for berry diameter, as well as the number of berries and grape bunches were given, from which a forecast of the true biological yield can be made. Additional yield parameters can potentially be derived from the 3D data, as well. The pipeline is holistic in that data acquisition, data interpretation and data quantification are covered. The results demonstrate the potential of the pipeline for future automated high-throughput, large-data phenotyping tasks in the field.

multiple

## Figures and Tables

**Table 1:** Grape bunch class recall and precision for the classification of the VSP trellis grapevine row. IVM, import vector machines.

Grape Bunch Class			
	# of True Grape Bunch Points	Recall (%)	Precision (%)
**IVM**—Initial classification	58,895	86.6	38.8
**GCO**—Label smoothing	58,895	94.0	62.8

**Table 2:** Grape bunch class recall and precision for the classification of the SMPH grapevine row.

Grape Bunch Class			
	# of True Grape Bunch Points	Recall (%)	Precision (%)
**IVM**—Initial classification	48,183	81.2	23.2
**GCO**—Label smoothing	48,183	88.7	38.4

**Table 3:** Grape bunch class recall and precision for SFH and HSV features.

Grape Bunch Class		
	Recall (%)	Precision (%)
**SFH**	76.9	71.4
**HSV**	47.2	86.1

**Table 4:** Grape bunch recall and precision for yield parameter estimation of a VSP trellis grapevine row.

Grape Bunch Parameter Accuracy		
	Recall (%)	Precision (%)
**Meter 1**	57	76
**Meter 2**	87	87
**Meter 3**	87	76
**Meter 4**	81	71
**Meter 5**	100	83
$\bar{Meter}$	82	79
*σ*	16	6

**Table 5:** Grape bunch recall and precision for yield parameter estimation of the SMPH grapevine row.

Grape Bunch Parameter Accuracy		
	Recall (%)	Precision (%)
**Meter 1**	85	81
**Meter 2**	79	90
**Meter 3**	67	100
**Meter 4**	80	100
**Meter 5**	75	86
$\bar{Meter}$	77	91
*σ*	7	9

**Table 6:** Berry recall and precision for the berry yield parameter on 10 grape bunches of a VSP trellis grapevine row.

	# of Berries in the Image	Recall (%)	Precision (%)
**Grape Bunch 1**	11	90.9	100
**Grape Bunch 2**	51	90.2	100
**Grape Bunch 3**	66	75.8	94.3
**Grape Bunch 4**	44	79.5	94.6
**Grape Bunch 5**	20	45.0	100
**Grape Bunch 6**	35	86.6	100
**Grape Bunch 7**	42	81.0	97.1
**Grape Bunch 8**	97	80.4	94.0
**Grape Bunch 9**	100	81.0	97.6
**Grape Bunch 10**	44	84.1	100
$\bar{Mean}$		77.6	97.8
*σ*		13.2	2.6

**Table 7:** Berry recall and precision for berry yield parameter estimation on 10 grape bunches of the SMPH grapevine row.

	# of Berries in the Image	Recall (%)	Precision (%)
**Grape Bunch 1**	33	61.8	91.3
**Grape Bunch 2**	33	75.8	100
**Grape Bunch 3**	21	81.0	94.4
**Grape Bunch 4**	27	62.1	100
**Grape Bunch 5**	26	78.6	100
**Grape Bunch 6**	31	90.3	100
**Grape Bunch 7**	24	70.8	94.4
**Grape Bunch 8**	24	91.7	100
**Grape Bunch 9**	25	88.0	95.7
**Grape Bunch 10**	29	72.4	100
$\bar{Mean}$		77.2	97.6
*σ*		10.8	3.3

**Table 8:** Final number of berries and berry diameter estimation for 5 m of both rows.

	# of Estimated Berries	Estimated Mean Berry Diameter (mm)	Measured Mean Berry Diameter (mm)
VSP trellis row	1577	$\bar{13.7}\pm 1.7$	$\bar{11.8}\pm 1.4$
SMPH row	629	$\bar{12.8}\pm 1.6$	$\bar{10.4}\pm 1.6$
